# Drug-Drug Interactions, Safety, and Pharmacokinetics of EGFR Tyrosine Kinase Inhibitors for the Treatment of Non–Small Cell Lung Cancer

**Published:** 2018-03-01

**Authors:** Colleen R. Kucharczuk, Alex Ganetsky, J. Michael Vozniak

**Affiliations:** 1 Hospital of the University of Pennsylvania, Philadelphia, Pennsylvania;; 2 Hospital of the University of Pennsylvania, Philadelphia, Pennsylvania at the time of article submission, currently at Merck & Co., Inc, Kenilworth, New Jersey

## Abstract

Inhibitors of the epidermal growth factor receptor (EGFR) are important treatment options for non–small cell lung cancer (NSCLC) patients with activating *EGFR* mutations. Erlotinib, gefitinib, afatinib, and osimertinib are approved for use in NSCLC patients, and several other agents are in clinical development. The objectives of this article are to review the pharmacokinetic and known drug interaction data for EGFR tyrosine kinase inhibitors (TKIs) available for use in NSCLC patients, as well as adverse events (AEs) commonly observed with EGFR-TKI treatment, and to discuss relevant management strategies. The importance of this information for patient care is explored from the perspective of advanced practitioners. Pharmacokinetic, drug-interaction, and safety data are included for EGFR inhibitors approved for NSCLC (erlotinib, gefitinib, afatinib, and osimertinib). Relevant dose modifications and AE management strategies are also reviewed. The interdisciplinary health-care team plays an essential role in patient education, care planning, and medication administration. As such, it is essential that advanced practitioners understand the safety profiles and the potential for drug interactions with EGFR TKIs to ensure patients achieve the maximum benefit from these agents.

The identification of activating mutations in the epidermal growth factor receptor (EGFR) has expanded treatment options for non–small cell lung cancer (NSCLC), where the presence of these mutations can sensitize tumors to EGFR inhibitors (Rosell et al., 2010). For patients whose tumors have sensitizing *EGFR* mutations, EGFR tyrosine kinase inhibitors (TKIs) are important components of the NSCLC treatment landscape. Four EGFR TKIs are approved by the US Food and Drug Administration (FDA) for use in NSCLC patients (erlotinib [Tarceva], gefitinib [Iressa], afatinib [Gilotrif], and osimertinib [Tagrisso]), and several others are in development. A thorough understanding of the safety profiles and drug interactions of EGFR TKIs is critical for advanced practitioners, who have a key role in educating patients on their safe and effective use. Here, we review relevant pharmacokinetic (PK) data and known drug interactions for each of the FDA-approved EGFR TKIs. We also summarize the most common EGFR-TKI-associated adverse events (AEs) and discuss management strategies, highlighting the role of advanced practitioners in safely managing EGFR-TKI use to ensure maximum patient benefit.

## APPROVED EGFR TKIS

**Erlotinib**

**Figure 1:**
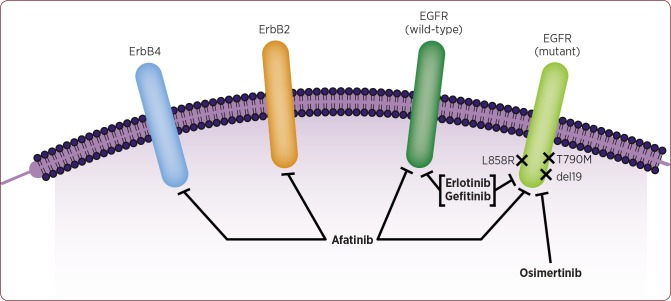
Mechanisms of action of approved EGFR TKIs for NSCLC. Erlotinib and gefitinib are reversible EGFR inhibitors that bind to both wild-type and mutant EGFR, including L858R and del19 forms. In contrast, afatinib irreversibly binds to wild-type and mutant EGFR, as well as to the ErbB family members ErbB2 and ErbB4. The recently approved, mutant-specific, EGFR inhibitor osimertinib binds preferentially to mutant forms of the receptor, particularly T790M. EGFR = epidermal growth factor receptor; TKI = tyrosine kinase inhibitor; NSCLC = non–small cell lung cancer; L858R = exon 21; del19 = deletion 19.

Erlotinib is an oral, reversible inhibitor of wild-type and mutant EGFR (Figure 1) indicated for the first-line treatment of metastatic NSCLC harboring deletion 19 (del19) or exon 21 (L858R) substitution EGFR mutations (OSI Pharmaceuticals, LLC, 2015). Erlotinib is also indicated for the treatment of locally advanced NSCLC after chemotherapy failure and for maintenance treatment of locally advanced or metastatic NSCLC that has not progressed after 4 cycles of platinum-based therapy (OSI Pharmaceuticals, LLC, 2015). The recommended erlotinib dose is 150 mg/day on an empty stomach, as PK studies have demonstrated that bioavailability is increased with food (Katsuya et al., 2015; OSI Pharmaceuticals, LLC, 2015). Additional PK analyses (Table 1) have shown that erlotinib is ~60% bioavailable, has a long half-life (> 36 hours), and is metabolized primarily by cytochrome P450 (CYP) enzymes, particularly CYP3A4, in the liver (Lu et al., 2006; OSI Pharmaceuticals, LLC, 2015).

**Table 1:**
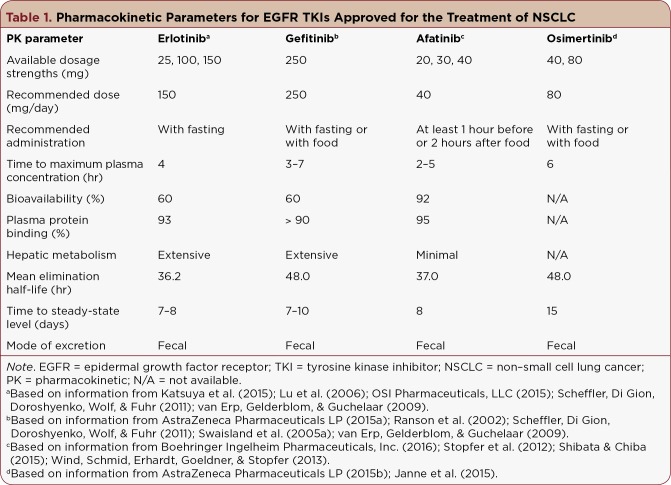
Pharmacokinetic Parameters for EGFR TKIs Approved for the Treatment of NSCLC

Generally, no significant effects on PK were observed with age, gender, or weight differences (Lu et al., 2006; OSI Pharmaceuticals, LLC, 2015), although one study (N = 55) demonstrated lower erlotinib exposure in African-American NSCLC patients (Phelps et al., 2014). Patients with mild or moderate hepatic impairment had similar PK as patients with normal liver function; thus, erlotinib dose modifications are not recommended for impaired hepatic function, but patients should be monitored closely (O’Bryant et al., 2012). Hepatotoxicity can occur with erlotinib, and patients with baseline hepatic impairment have increased risk. Periodic liver testing should be performed, and erlotinib should be withheld for total bilirubin levels greater than three times the upper limit of normal or transaminases greater than five times the upper limit of normal. No studies have been conducted in patients with renal failure, although a case study reported that erlotinib was tolerated in three NSCLC patients with chronic renal failure (Gridelli, Maione, Galetta, & Rossi, 2007). Accordingly, there are no dose modifications recommended for these patients (OSI Pharmaceuticals, LLC, 2015).

Erlotinib exposure may be affected by concomitant use of other drugs (Table 2). Drugs that decrease acid can decrease erlotinib exposure. Patients should avoid use of proton pump inhibitors, such as pantoprazole and omeprazole, while taking erlotinib due to potential effects on erlotinib concentration (Kletzl et al., 2015; OSI Pharmaceuticals, LLC, 2015; Ter Heine et al., 2010). When histamine H2-receptor antagonists (H2 antagonists), such as ranitidine, are used concomitantly, erlotinib should be given 10 hours after the H2 antagonist and ≥ 2 hours before the next antagonist dose (Kletzl et al., 2015; OSI Pharmaceuticals, LLC, 2015). Antacids should be administered several hours before or after erlotinib (Kletzl et al., 2015; OSI Pharmaceuticals, LLC, 2015). It is important that these recommendations are followed, as decreased erlotinib efficacy was reported in NSCLC patients using gastric acid–suppressing medications (Chu et al., 2015). Alternate means of managing gastroesophageal reflux disease include sucralfate and limiting gastric irritants and foods high in acidity.

**Table 2:**
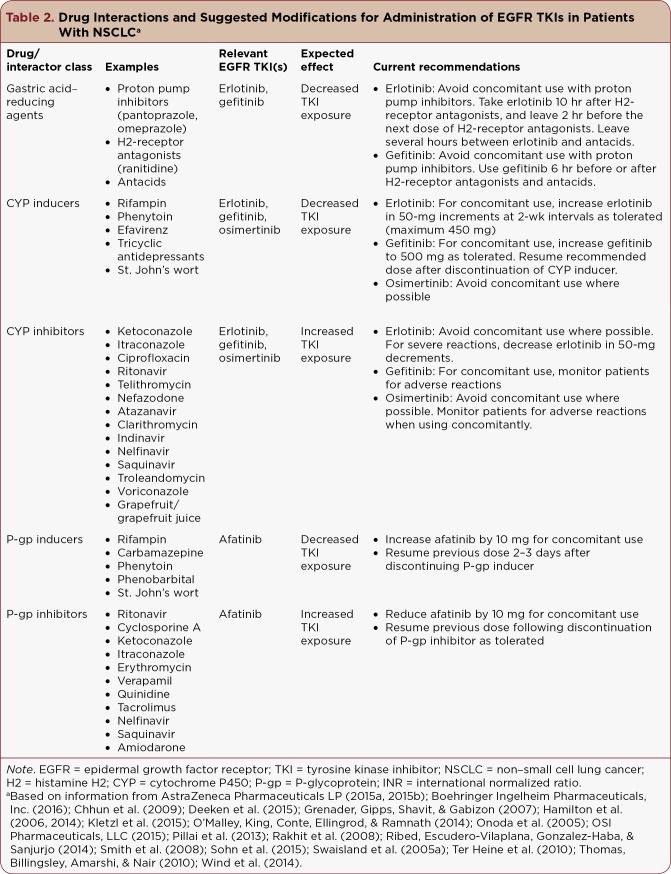
Table 2. Drug Interactions and Suggested Modifications for Administration of EGFR TKIs in Patients With NSCLCa

**Table 2:**
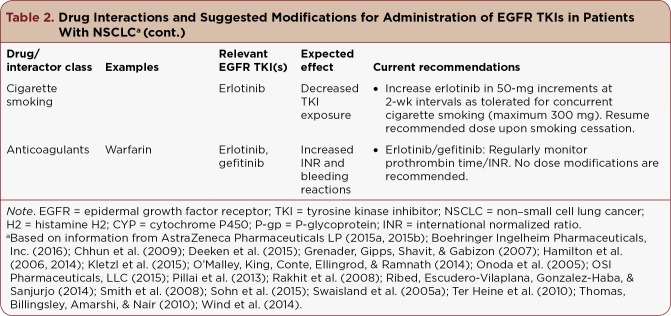
Table 2. Drug Interactions and Suggested Modifications for Administration of EGFR TKIs in Patients With NSCLCa (cont.)

Due to its extensive metabolism by CYP3A4, erlotinib may be affected by other drugs that enhance or inhibit CYP activity or undergo CYP-mediated metabolism (Table 2). Advanced practitioners should be aware if patients are taking CYP inhibitors, including certain antifungal, antibiotic, and antiretroviral medications, as these agents can increase erlotinib exposure (Deeken et al., 2015; Pillai et al., 2013; Rakhit et al., 2008). Additionally, grapefruit or grapefruit juice are CYP inhibitors and may increase erlotinib plasma concentrations (Smith et al., 2008). Likewise, patients should avoid concomitant use of erlotinib and CYP inducers, such as rifampin, phenytoin, and St. John’s wort, as their use may affect drug exposure (Deeken et al., 2015; Grenader, Gipps, Shavit, & Gabizon, 2007; Hamilton et al., 2014; Pillai et al., 2013). Use of erlotinib with dexamethasone, also a CYP inducer, resulted in a 0.6-fold decrease in erlotinib exposure and may require dose escalation up to the maximum of 450 mg daily (Deeken et al., 2015). For situations where concomitant use of erlotinib with CYP interactors is necessary, erlotinib dose should be decreased (CYP inhibitors) or increased (CYP inducers) in 50-mg decrements/increments at 2-week intervals as tolerated (Table 2).

Drug interactions may also occur between erlotinib and drugs cleared by uridine diphosphate–glucuronosyltransferases, organic cation transporters (OCTs), and organic anion transporters (OATs), potentially affecting exposure to drugs that are substrates of these transporters (e.g., metformin, hydroxyurea, methotrexate, certain statins; Johnston, Rawling, Chan, Zhou, & Murray, 2014; Liu, Ramirez, House, & Ratain, 2010; Minematsu & Giacomini, 2011).

Erlotinib exposure also can be decreased by cigarette smoking (Hamilton et al., 2006; O’Malley, King, Conte, Ellingrod, & Ramnath, 2014; Sohn et al., 2015). Current smokers, defined as smoking a minimum of 10 cigarettes per day for at least 1 year, required double the dose (300 mg) of erlotinib as nonsmokers (150 mg) to achieve therapeutic concentrations of the drug (Hamilton et al., 2006). Median survival times for patients receiving erlotinib are longest for those who never smoked compared with former smokers and current smokers (O’Malley et al., 2014). Erlotinib dose increases of 50 mg are advised at 2-week intervals to a maximum of 300 mg daily for concurrent cigarette smoking (OSI Pharmaceuticals, LLC, 2015). The interdisciplinary team of health-care providers can be instrumental in smoking cessation education; once cigarette smoking has ceased, the recommended erlotinib dose (100 or 150 mg daily) can be immediately resumed (Table 2).

Adverse interactions between erlotinib and coumarin-based anticoagulants, such as warfarin, have been observed, including increased international normalized ratios (INRs) and bleeding reactions (Thomas, Billingsley, Amarshi, & Nair, 2010). Patients using warfarin should be monitored closely for changes in INR while using erlotinib (Table 2).

**Gefitinib**

Gefitinib is another reversible inhibitor of wild-type and mutant EGFR (Figure 1) indicated for the first-line treatment of patients with metastatic NSCLC whose tumors have del19 or L858R EGFR mutations (AstraZeneca Pharmaceuticals LP, 2015a). Gefitinib should be taken orally at a dose of 250 mg/day with or without food (AstraZeneca Pharmaceuticals LP, 2015a). The PK profile of gefitinib is similar to that of erlotinib, but gefitinib has a longer half-life of 48 hours (Ranson et al., 2002; Table 1); gefitinib availability is not significantly influenced by food (Swaisland et al., 2005a). Patient age, weight, gender, or ethnicity do not have any apparent effect on gefitinib PK (AstraZeneca Pharmaceuticals LP, 2015a; Li et al., 2006). Gefitinib PK were shown to be impacted by hepatic impairment due to cirrhosis but not liver metastases (Horak et al., 2011), and patients with moderate to severe hepatic impairment should be monitored for an increased risk of adverse reactions (AstraZeneca Pharmaceuticals LP, 2015a). Dose modifications are recommended based on results of periodic liver testing: for grade 2 or higher transaminase elevations, gefitinib should be withheld, and for severe hepatic impairment, treatment should be discontinued. No studies have been conducted to evaluate gefitinib PK in patients with renal impairment.

Due to their similar structure and PK profile, gefitinib and erlotinib share similar drug-interaction potential. Like erlotinib, gefitinib metabolism occurs mainly through CYP enzymes in the liver, including CYP3A4 and CYP2D6 (Li, Zhao, He, Hidalgo, & Baker, 2007). CYP inhibitors (e.g., itraconazole) may increase gefitinib plasma levels, and patients taking gefitinib with CYP inhibitors should be monitored closely (Swaisland et al., 2005b). For concomitant use with strong CYP inducers, such as phenytoin and rifampin, which can decrease gefitinib levels (Chhun et al., 2009; Swaisland et al., 2005b), gefitinib daily dose can be increased from 250 mg to 500 mg. Patients should resume the recommended dose of 250 mg after discontinuation of the CYP inducer (AstraZeneca Pharmaceuticals LP, 2015a). Gefitinib can inhibit the activity of drug transporters, such as OCTs (metformin, oxaliplatin) and OATs (montelukast, certain statins, sulfated estrogens, thyroxine), potentially altering the exposure of coadministered medications that are substrates for these transporters (Johnston et al., 2014; Minematsu & Giacomini, 2011).

Like erlotinib, acid-reducing medications may decrease gefitinib exposure. Gefitinib should not be used with proton pump inhibitors and, when concomitant use is necessary, dosing of gefitinib and the proton pump inhibitor should be spaced by 12 hours (AstraZeneca Pharmaceuticals LP, 2015a). When treatment with antacids or H2 antagonists is required, gefitinib should be taken 6 hours before or after the acid-reducing agent (AstraZeneca Pharmaceuticals LP, 2015a). Similar to erlotinib, gefitinib use has been linked to adverse reactions, including hemorrhage and INR elevations, in patients using warfarin or other coumarin derivatives (Onoda et al., 2005; Ribed, Escudero-Vilaplana, Gonzalez-Haba, & Sanjurjo, 2014), and therefore these patients should be monitored closely for potential reactions.

**Afatinib**

Afatinib is an oral, irreversible ErbB family blocker that targets mutant and wild-type EGFR/ErbB1, human epidermal growth factor receptor (HER) 2/ErbB2, and HER4/ErbB4, which results in the inhibition of HER3/ErbB3 phosphorylation (Li et al., 2008; Solca et al., 2012; Figure 1). It is currently indicated for the first-line treatment of patients with metastatic NSCLC whose tumors harbor del19 or L858R EGFR mutations and for metastatic squamous NSCLC progressing after platinum therapy (Boehringer Ingelheim Pharmaceuticals, 2016). The recommended afatinib dose is 40 mg once daily, taken ≥ 1 hour before or 2 hours after a meal (Boehringer Ingelheim Pharmaceuticals, 2016).

The PK profile of afatinib is presented in Table 1 (Wind, Schmid, Erhardt, Goeldner, & Stopfer, 2013). Weight, age, gender, race, and mild or moderate hepatic impairment do not have a significant effect on afatinib PK (Freiwald et al., 2014; Schnell et al., 2014). Severe renal impairment has been shown to increase afatinib exposure; thus, according to the US prescribing information, these patients should receive a starting dose of 30 mg afatinib (Boehringer Ingelheim Pharmaceuticals, 2016). Afatinib is minimally metabolized by hepatic enzymes (Shibata & Chiba, 2015; Stopfer et al., 2012); therefore, CYP-interacting agents are unlikely to impact afatinib PK. No dose modifications are recommended for patients with mild (Child-Pugh A) or moderate (Child-Pugh B) hepatic impairment (Boehringer Ingelheim Pharmaceuticals, 2016). No studies have examined the effects of afatinib on patients with severe hepatic impairment (Child-Pugh C), although these patients should be closely monitored and have their dose adjusted for tolerability (Boehringer Ingelheim Pharmaceuticals, 2016). If periodic liver testing shows worsening of liver function, gefitinib should be withheld; for those who develop severe hepatic impairment, treatment should be discontinued.

Afatinib is a substrate and inhibitor of the P-glycoprotein (P-gp) drug transporter, and inhibitors and enhancers of P-gp activity may impact afatinib exposure. One study demonstrated that concomitant administration of afatinib with ritonavir, a P-gp inhibitor, increased afatinib exposure, although this increase was minimized by spacing out the medications by 6 hours (Wind et al., 2014). When concomitant use of afatinib with P-gp inhibitors is required, the afatinib dose should be reduced by 10 mg, as tolerated (Boehringer Ingelheim Pharmaceuticals, 2016; Table 2). Additionally, coadministration of afatinib with the P-gp inducer rifampin decreased afatinib plasma concentrations (Wind et al., 2014). Patients taking afatinib with a P-gp inducer should increase their afatinib dose by 10 mg (Boehringer Ingelheim Pharmaceuticals, 2016; Table 2). There is also evidence for an inhibitory effect of afatinib on OATs, although this effect is less potent than that observed with erlotinib or gefitinib (Johnston et al., 2014).

**Osimertinib**

Osimertinib was approved for metastatic NSCLC patients whose tumors harbor the T790M *EGFR* mutation following progression on EGFR-TKI therapy in 2015 (AstraZeneca Pharmaceuticals LP, 2015b; Kuiper et al., 2014). Osimertinib inhibits mutant forms of EGFR, including T790M, del19, and L858R, with minimal inhibition of wild-type EGFR protein (Cross et al., 2014; Figure 1). The recommended osimertinib dose is 80 mg once daily, taken orally with or without food (AstraZeneca Pharmaceuticals LP, 2015b).

There are limited PK and drug interaction data available for osimertinib, as its approval was recent and studies are ongoing. Pharmacokinetic data suggest a long half-life and relatively longer time to maximum plasma concentration compared with other approved EGFR TKIs (AstraZeneca Pharmaceuticals LP, 2015b; Jänne et al., 2015; Table 1). Based on its metabolism by CYP3A enzymes, osimertinib exposure may be impacted by CYP enzyme inducers or inhibitors, although extensive studies have not yet been performed (AstraZeneca Pharmaceuticals LP, 2015b). Patients taking osimertinib should avoid concomitant use of strong CYP inhibitors or inducers, and patients should be closely monitored when coadministration is necessary (AstraZeneca Pharmaceuticals LP, 2015b; Table 2).

## SAFETY CONSIDERATIONS WITH EGFR-TKI THERAPY

EGFR TKIs have been associated with specific AEs, including diarrhea, mucositis, rash, and paronychia (Melosky & Hirsh, 2014). Because the incidence of these AEs can be high with EGFR-TKI use, dose optimization (where applicable) can mitigate their effects. Advanced practitioners can help anticipate and effectively manage these AEs so that patients can remain on therapy and maximize the clinical benefits of EGFR TKIs.

**Diarrhea**

Diarrhea is one of the most commonly observed AEs with EGFR TKIs (Melosky & Hirsh, 2014) and is graded from 1 to 5 based on frequency and severity (U.S. Department of Health and Human Services, National Institutes of Health, & National Cancer Institute, 2010). Grade 1 is defined as an increase of less than 4 stools per day over baseline, grade 2 as 4 to 6 stools per day over baseline, grade 3 as more than 7 stools per day over baseline or incontinence, grade 4 includes life-threatening consequences, and grade 5 is death (Cancer.net, 2017). In clinical trials supporting the approval of EGFR TKIs, incidence of any grade diarrhea ranged from 30.8% with gefitinib to 95.2% with afatinib (Douillard et al., 2014; Jänne et al., 2015; Rosell et al., 2012; Sequist et al., 2013). The incidence of grade ≥ 3 diarrhea was ≤ 5% for erlotinib, gefitinib, and osimertinib and ranged from 5.4% to 14.4% with afatinib when starting at 40 mg (Douillard et al., 2014; Jänne et al., 2015; Rosell et al., 2012; Sequist et al., 2013; Wu et al., 2014). Management strategies for diarrhea include dietary modifications such as avoiding foods that are difficult to digest (e.g., broccoli, cabbage) and following the bananas, rice, applesauce, and toast (BRAT) diet until symptoms begin to resolve (Melosky & Hirsh, 2014). Loperamide can also be used to treat diarrhea; 4 mg should be given after the first episode of diarrhea, and 2 mg can be administered every 2 to 4 hours until diarrhea stops, typically not exceeding 16 mg/day for prescription use (Drugs.com, 2016; Hirsh, 2011; Melosky & Hirsh, 2014; Sipples, Andan, & Eaby-Sandy, 2015). For more severe diarrhea, diphenoxylate and atropine or octreotide can be considered. The EGFR TKI should be discontinued until severe diarrhea resolves, at which point the EGFR TKI can often be reintroduced (Hirsh, 2011; Melosky & Hirsh, 2014).

**Rash**

**Figure 2:**
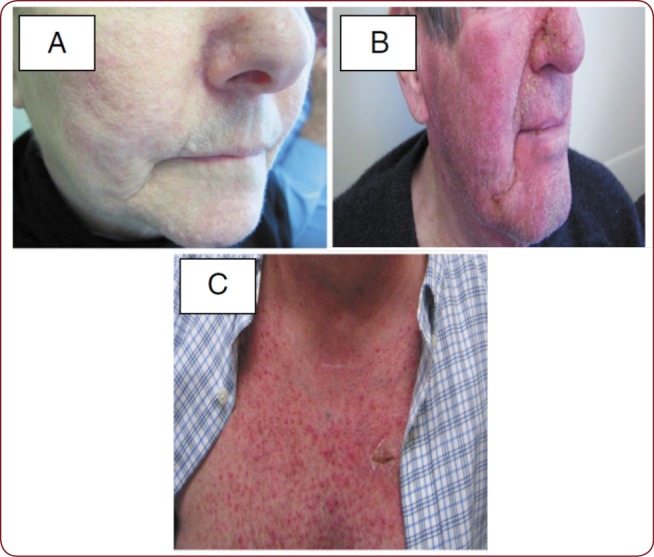
Mild papulopustular (acneiform) rash (A), and papulopustular (acneiform) rash (B and C).

Dermatologic AEs, such as rash and acne, are also commonly observed with EGFR TKIs, and patient education should be provided at the initiation of therapy (Figure 2). Reported rates for these AEs in clinical trials were between 40.0% and 89.1% for rash of any grade and up to 16.2% for grade ≥ 3 rash (Douillard et al., 2014; Jänne et al., 2015; Rosell et al., 2012; Sequist et al., 2013). Rashes typically present in stages, with early erythema and edema observed in weeks 1 to 2 of treatment. In week 2, papulopustular eruptions can occur in skin with a high density of sebaceous glands, followed by crusting of the skin in week 4 (Lacouture et al., 2011). Over the next 4 to 6 weeks, patients can experience dry skin and erythema (Lacouture & Melosky, 2007). Based on data from randomized controlled trials, preventive therapy is recommended using 1% hydrocortisone, alcohol-free emollient creams, sunscreen, and doxycycline 100 mg twice daily (Hirsh, 2011; Lacouture et al., 2011; Melosky & Hirsh, 2014). Additional treatments for EGFR-TKI-associated rash include medium- to high-potency topical corticosteroids and isotretinoin at doses lower than those used for acne (Lacouture et al., 2011). EGFR-TKI treatment should be discontinued for severe reactions and can often be reintroduced at a modified dose upon resolution of the rash (Hirsh, 2011; Melosky & Hirsh, 2014).

**Stomatitis/Mucositis**

Stomatitis (inflammation of the mouth) and mucositis (inflammation of the gastrointestinal tract) have been observed with EGFR-TKIs (Melosky & Hirsh, 2014; Soria et al., 2015). In the LUX-Lung 3 trial, stomatitis/mucositis was seen in 72.1% of patients receiving afatinib, with 8.7% experiencing grade ≥ 3 stomatitis/mucositis (Sequist et al., 2013). Grades 1 and 2 mucosal inflammation were also reported in 12% of patients receiving osimertinib, 18% receiving erlotinib (1% grade ≥ 3), and 7% receiving gefitinib (0.3% grade ≥ 3) (AstraZeneca Pharmaceuticals LP, 2015a; Jänne et al., 2015; OSI Pharmaceuticals, LLC, 2015).

Suggested management strategies for stomatitis/mucositis include maintaining good oral hygiene with alcohol-free products, triamcinolone acetonide, clobetasol, erythromycin, viscous lidocaine, and magic mouthwash (Melosky & Hirsh, 2014; Sipples et al., 2015). Analgesics may also be temporarily required for pain management associated with stomatitis/mucositis (Harris, 2006). As with other EGFR-TKI-associated AEs, it is suggested that treatment with the EGFR TKI be paused until severe cases of stomatitis/mucositis are resolved or improved. Following improvement of the AE, the EGFR TKI can be reintroduced at a lower dose (Melosky & Hirsh, 2014).

**Paronychia**

Another AE seen more frequently with afatinib and osimertinib is paronychia, an infection of the tissue where the skin meets the nail, which can often be tender and painful. Paronychia was reported in 56.8% of patients receiving afatinib, with grade ≥ 3 paronychia observed in 11.4% (Sequist et al., 2013). Paronychia was reported in 17% of patients taking osimertinib, with less than 0.5% of cases reported as grade ≥ 3 (Jänne et al., 2015). Recommended strategies for managing paronychia include the use of topical clobetasol or antibiotics/antiseptics, vinegar soaks, silver nitrate, and nail avulsion for severe cases (Melosky & Hirsh, 2014; Sipples et al., 2015).

**Dose-Modification Protocols for Adverse Events**

Dose modifications are recommended for patients taking EGFR TKIs who experience grade ≥ 3 AEs, particularly diarrhea and rash. These modifications generally involve withholding the TKI until AE improvement, at which time the TKI can be reintroduced at a lower dose (AstraZeneca Pharmaceuticals LP, 2015a, 2015b; OSI Pharmaceuticals, LLC, 2015). A dose-optimization scheme has been recommended for patients taking afatinib who experience drug-related grade 3 and certain prolonged grade 2 AEs (diarrhea persisting 2 or more consecutive days despite antidiarrheal medication, cutaneous reactions lasting more than 7 days or that is intolerable, and renal impairment; Sequist et al., 2013; Sipples et al., 2015; Yang et al., 2016). Patients should discontinue afatinib temporarily, and appropriate supportive care measures should be implemented. Upon AE improvement, afatinib can be reintroduced at a dose 10 mg lower than the previous dose. Importantly, progression-free survival was similar for patients who dose-reduced on afatinib vs. those who did not reduce their dose, suggesting that dose modification can allow patients to manage AEs without compromising afatinib efficacy (Yang et al., 2016).

## DISCUSSION AND HEALTH-CARE TEAM PERSPECTIVES

EGFR TKIs are a key component of the NSCLC treatment landscape, and familiarity with these agents is critical for achieving maximum treatment benefits for patients. Health-care team members are responsible for understanding the drug profile of medications they are prescribing or administering, are key informants to patients, and are crucial to ensuring patient safety and adherence. Comprehensive assessment of patients taking EGFR TKIs helps to identify and quantify adverse reactions and contributes to increased medication compliance. Furthermore, advanced practitioners are pivotal to the interdisciplinary team, help to quantify the impact of cancer treatments on patients’ quality of life, and modify treatment care plans as necessary to meet the needs of their patients and their treatment outcomes.

The health-care team provides patient education about EGFR TKIs and can encourage open communication with patients about concurrent medication use and onset of side effects. Providing education can help keep patients out of the hospital due to uncontrolled AEs and can reduce health-care spending by preventing avoidable hospitalizations (Brooks et al., 2014; Newcomer, 2014). Effective counseling and education empowers the patient to deal with EGFR-TKI-associated side effects, and advanced practitioners are an important resource to patients and families on how to manage and mitigate EGFR-TKI-associated toxicities. Continued understanding of the anticipated drug-interaction and side-effect profiles of targeted therapies is the key to keeping patients on the proper doses of these medications to maximize benefit.

**Acknowledgment**

The authors received no direct compensation related to the development of the manuscript. Writing, editorial support, and formatting assistance were provided by Lauren Fink, PhD, of MedErgy, which was contracted and funded by Boehringer Ingelheim Pharmaceuticals, Inc. (BIPI). BIPI was given the opportunity to review the manuscript for medical and scientific accuracy, as well as intellectual property considerations.
